# Impact of real-time angiographic co-registered optical coherence tomography on percutaneous coronary intervention: the OPTICO-integration II trial

**DOI:** 10.1007/s00392-020-01739-1

**Published:** 2020-09-05

**Authors:** Vera S. Schneider, Felix Böhm, Katharina Blum, Matthias Riedel, Youssef S. Abdelwahed, Jens Klotsche, Julia K. Steiner, Andrea Heuberger, Carsten Skurk, Hans-Christian Mochmann, Alexander Lauten, Georg Fröhlich, Ursula Rauch-Kröhnert, Arash Haghikia, David Sinning, Barbara E. Stähli, Ulf Landmesser, David M. Leistner

**Affiliations:** 1 Department of Cardiology, University Heart Center Berlin and Charité University Medicine Berlin, Campus Benjamin-Franklin (CBF), 12203 Berlin, Germany; 2 DZHK (German Centre for Cardiovascular Research) Partner Site Berlin, 12203 Berlin, Germany; 3 Berlin Institute of Health (BIH), 10117 Berlin, Germany; 4 German Rheumatism Research Center Berlin, and Institute for Social Medicine, Epidemiology Und Heath Economy, Charite University Medicine Berlin, Campus Charité Mitte, 10117 Berlin, Germany

**Keywords:** OCT, Angiographic co-registration, Geographic mismatch, Edge dissection, PCI

## Abstract

**Aims:**

Longitudinal geographic mismatch (LGM) as well as edge dissections are associated with an increased risk of adverse events after percutaneous coronary intervention (PCI). Recently, a novel system of real-time optical coherence tomography (OCT) with angiographic co-registration (ACR) became available and allows matched integration of cross-sectional OCT images to angiography. The OPTICO-integration II trial sought to assess the impact of ACR for PCI planning on the risk of LGM and edge dissections.

**Methods:**

A total of 84 patients were prospectively randomized to ACR-guided PCI, OCT-guided PCI (without co-registration), and angiography-guided PCI. Primary endpoint was a composite of major edge dissection and/or LGM as assessed by post-PCI OCT.

**Results:**

The primary endpoint was significantly reduced in ACR-guided PCI (4.2%) as compared to OCT-guided PCI (19.1%; *p* = 0.03) and angiography-guided PCI (25.5%; *p* < 0.01). Rates of LGM were 4.2%, 17.0%, and 22.9% in the ACR-guided PCI, in the OCT-guided PCI, and the angiography-guided PCI groups, respectively (ACR vs. OCT *p* = 0.04; ACR vs. angiography *p* = 0.04). The number of major edge dissections was low and without significant differences among the study groups (0% vs. 2.1% vs. 4.3%).

**Conclusion:**

This study for the first time demonstrates superiority of ACR-guided PCI over OCT- and angiography-guided PCI in reducing the composite endpoint of major edge dissection and LGM, which was meanly driven by a reduction of LGM.

**Graphical abstract:**

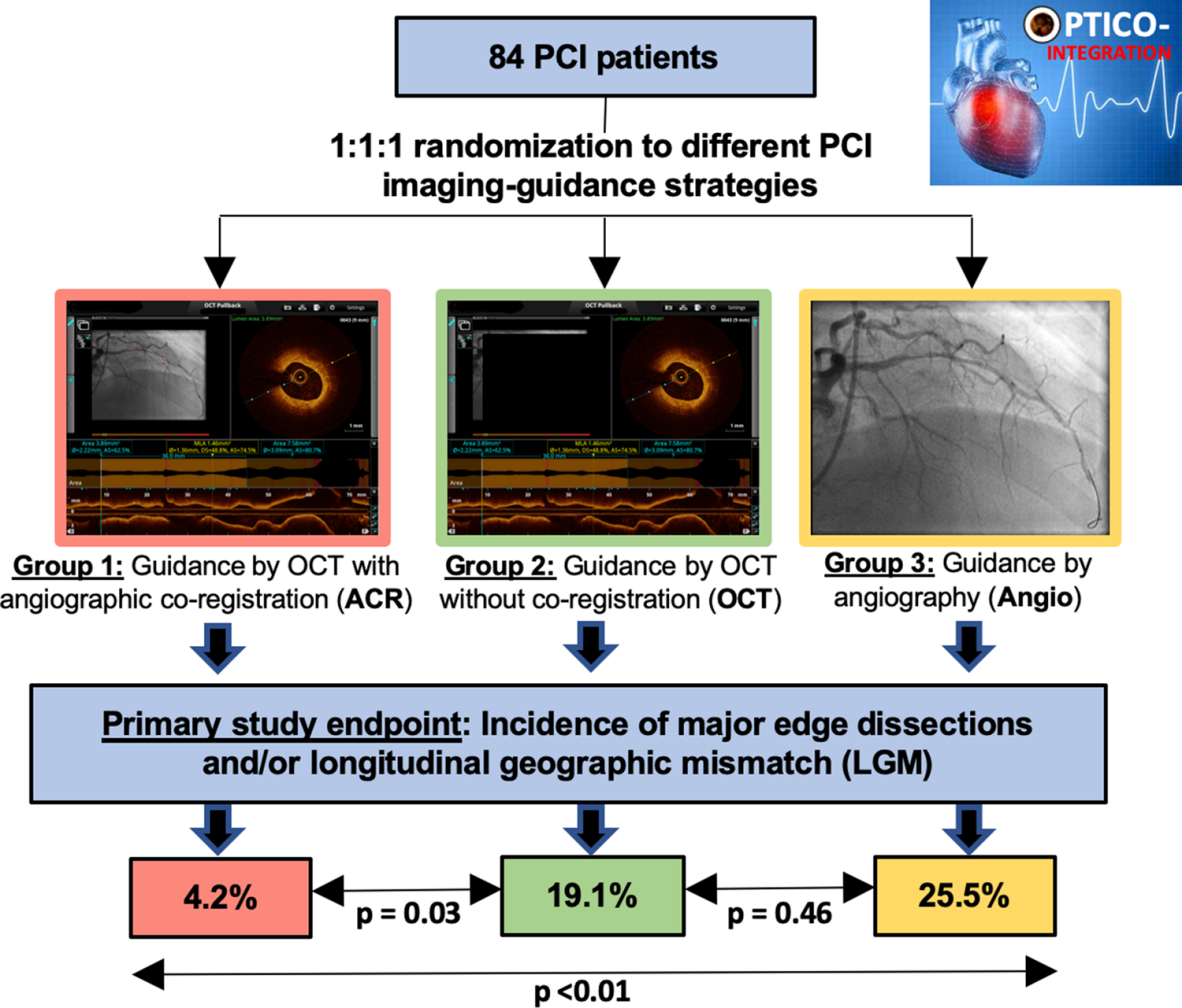

## Introduction

Percutaneous coronary intervention (PCI) has become standard therapy for chronic coronary syndrome (CCS) [1] and shows favorable results using third-generation drug-eluting stents (DES) [2]. However, previous studies revealed residual disease at the stent edge—termed as longitudinal geographic mismatch (LGM)—and stent edge dissections to be associated with an increased risk for major adverse cardiac events (MACE) after PCI [3]. Optical-coherence-tomography (OCT)-guided PCI may reduce these complications by allowing a more detailed pre-procedural coronary lesion evaluation [3–6]. However, the use of OCT in this setting is limited by difficulties in matching cross-sectional OCT images to angiography, i.e., translating the optimal stent landing zone from OCT to angiography. Recently, a system for real-time co-registration of OCT images with angiography (ACR = angiographic co-registration) became available [7]. This technique may overcome these limitations and, therefore, strengthen the use of OCT for PCI planning. Recently, the OPTICO-Integration-I trial demonstrated, that ACR had significant impact on the selected PCI strategy, particularly in complex lesions [8]. However, the efficacy of ACR to improve PCI results as compared to OCT- and angiographic-guided PCI has never been evaluated.

Therefore, the “Impact of Real-time Angiographic Co-registered OCT on PCI Results” (OPTICO-Integration-II trial; NCT03646097) trial was designed as a prospective, randomized pilot study to evaluate the effect of ACR guided PCI compared to OCT- and angiographic-guided PCI on the incidence of LGM and/or stent edge dissections after PCI.

## Methods

### Study design

The OPTICO-Integration-II trial was a prospective, single-center, randomized, open label trial. Consecutive patients undergoing coronary angiography were screened for suitability at the Charité—University Medicine Berlin, Germany, between August 2017 and July 2018. Subjects were considered eligible when angiography revealed significant coronary artery disease (based on visual assessment in at least one native coronary artery together with a positive ischemia test in stable coronary artery disease or lesions causing acute coronary syndromes) [9]. Exclusion criteria comprised in-stent restenosis, ST-segment elevation acute coronary syndromes (STE-ACS), cardiogenic shock, left ventricular ejection fraction (LVEF) < 30%, estimated creatinine clearance (eGFR) < 40 ml/min, neoplasia on treatment or without a curative therapeutic approach, life expectancy < 24 months, pregnancy, participation in another investigational clinical trial, as well as inability or unwillingness to give written informed consent. Furthermore, lesions unsuitable for OCT imaging were excluded, such as severely calcified or extremely tortuous vessels, as well as very distal lesions or a reference vessel diameter of > 4 mm or < 2 mm. Finally, patients were randomly assigned in a 1:1:1 ratio to either ACR (group I), OCT (group II) or angiography-guided PCI (group III). Permuted block randomization with variable block size was performed by means of sealed opaque envelopes containing a computer-generated sequence. Randomization was conducted after crossing the target lesion with the coronary guidewire.

The study was approved by the local Ethics Committee at Charité—University Medicine Berlin, Germany (EA1/072/17) and was registered at ClinicalTrials.gov (NCT03646097). Written informed consent was obtained from all participating subjects.

### OCT and procedural details

For OCT imaging the OPTIS™ integrated system (St. Jude Medical, St. Paul, MN) with Dragonfly™ Duo (St. Jude Medical, St. Paul, MN, USA) catheters were used. After placement of a standard 0.014 inch coronary guidewire over a 6F guiding catheter and injection of 200 mg of intracoronary nitroglycerin an automatic OCT pullback was performed with a contrast flush (IMERON^®^ 350; Bracco Imaging Deutschland GmbH, Konstanz, Germany) using an automated power injector. OCT images covering the stenotic area and at least 10 mm of the vessel segments proximal and distal to the lesion and were recorded with an axial resolution of 200 μm.

For study groups I (ACR) and II (OCT), the OCT scan was immediately evaluated by the operator using the regular OCT acquisition console (OPTIS™ integrated system): In group I (ACR) the commercially available semi-automatic co-registration OPTIS™ integrated system was used for co-registration of coronary angiography and OCT [7]. This results in a matched side-by-side view of angiography and OCT was available for the operator, with a small white marker projected over the angiogram and indicating the exact localization (error margin about 1 mm [7]) of the displayed OCT frame on the angiogram. However, in study groups I (ACR) and II (OCT), operators were encouraged to choose a stent-landing zone, which ensures optimal target lesion coverage defined as untreated minimal lumen area (MLA) ≤ 60% of the adjacent reference segment lumen area up to 10 mm from the proximal and distal stent edges [5]. Furthermore, vessel-wall characteristics as plaque load were taken into account for selection of the optimal stent-landing zone. Stent size selection included assessment of the relation to the reference vessel size. Importantly, PCI optimization in the ACR and OCT group followed the ILUMIEN IV—OPTIMAL PCI OCT criteria (NCT03507777). Stent optimization in the angiography-guided PCI group was performed at the discretion of the operator. Details of the PCI strategy including need for pre-dilation, special lesion debarking, vessel reference diameter, intended lesion and stent length, intended stent diameter, intended stenting strategy and device landing zone were documented in every case.

After angiography-based stent optimization PCI results were documented by OCT in all three study groups to assess the primary study endpoint. After primary study endpoint evaluation, further post-PCI optimization was allowed based on the final OCT in all study groups according to individual operator‘s estimation. All OCT scans were post-hoc analyzed by an independent OCT core lab blinded for the randomization group using an external OCT analysis console (OPTIS™, St. Jude Medical, St. Paul, MN, USA) and performing Quantitative coronary angiography (QCA) for all lesions among the three study groups. Only very experienced operators (*n* = 5; > 1000 PCIs lifelong) were involved in this trial.

### Endpoints

The primary study endpoint was a composite imaging endpoint including major edge dissections and/or LGM as assessed by post-procedural OCT. Major edge dissections were defined as each dissection with flap angle ≥ 60° of the circumference of the vessel at the site of dissection and/or ≥ 3 mm in length after PCI according to the ILUMIEN IV—OPTIMAL PCI OCT criteria (NCT03507777). Longitudinal geographic mismatch (LGM) was defined as every untreated plaque with a minimal lumen area < 4.5mm^2^ within 5 mm of the reference segment. If the segment of the lesion was fully covered and the stent protruded maximal 5 mm beyond the predetermined landing zone in the post-PCI OCT analysis it was considered as no geographic mismatch, again in line to ILUMIEN IV criteria. Secondary study endpoints included each individual component of the primary endpoint as well as the incidence of any edge dissection (any visible edge dissection < 60 degrees of the circumference of the vessel and < 3 mm in length), stent underexpansion [proximal minimal stent area (MSA) < 90% of the proximal reference lumen area, and/or distal MSA < 90% of the distal reference lumen area] and stent malapposition (stent struts separated from the vessel wall with a distance from the adjacent intima of ≥ 0.2 mm and not associated with any side branch). Furthermore, PCI result was assessed by determining MSA, length of the stented segment, number of implanted stents, as well as procedure time, fluoroscopy time, contrast volume and complications during hospital stay.

### Statistical analysis

Sample size calculation was based on a retrospective evaluation of OCT-guided PCI cases (*n* = 350) within the Charite University Medicine Berlin using a system without ACR, where LGM was detectable in 35% and additional major edge dissections in 5% of all PCI cases. Under the assumption that a combined imaging endpoint including major edge dissections and/or LGM is detectable in 35% of the segments with OCT-guided PCI and can be markedly reduced to 5% of patients by ACR-guided PCI, a total of 48 patients (24 per group) yield a power of 80% (with a 2-sided *α* = 0.05) to detect a difference in the primary endpoint between patients with ACR-guided (study group I) and OCT-guided PCI (study group II). Furthermore, an equal group (*n* = 24) who underwent angiography-guided PCI (study group III) was included as an additional control group. Anticipating a drop-out rate of 12%—again based on retrospective image analysis at the study center—a total of 84 patients were considered sufficient. Given the fact that the primary endpoint can occur twice per patient, at the proximal and the distal stent edge, an edge-based analysis was performed to assess differences regarding the endpoints between groups.

All data were analyzed according to the intention-to-treat principle. Categorical variables were expressed as numbers and percentages, and continuous variables as means and standard deviation or medians and interquartile range (IQR). Categorical variables were assessed by the Chi square test or the Fisher’s exact test as appropriate. Continuous variables were tested for normal distribution using the Kolmogorov–Smirnov test with Lilliefors correction, and group comparisons were performed using the Kruskal–Wallis tests or analysis of variance (ANOVA). Stent-edge based comparisons among the three study groups were performed using multilevel mixed-effects logistic regression. All variables that were significant in univariate analysis were entered into the multivariate model. A *p* value of 0.05 was considered statistically significant. All statistical analyses were performed by SPSS Version 22 (IBM Corp., Armonk, USA).

## Results

### Baseline characteristics

**Fig. 1:**
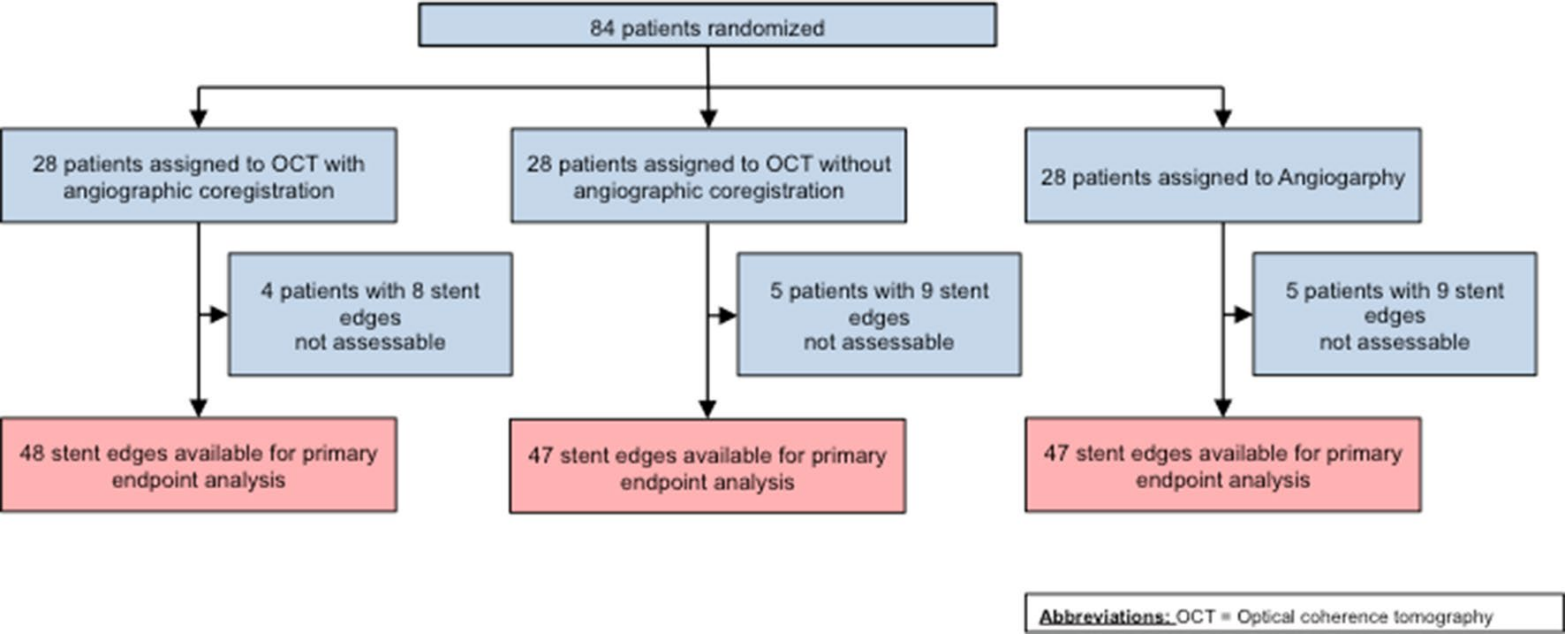
Study flow. Due to image quality issues some stent edges were not assessable in the respective groups of the trial. For the final analysis, a total of 48 stent edges in the ACR group, 47 stent edges in the OCT group and 47 stent edges in the angiography group were available for primary endpoint analysis. OCT denotes optical coherence tomography

Of 198 screened patients, a total of 84 patients were included and randomly assigned to undergo PCI guided by ACR (group I), OCT (group II) or angiography only (group III). The study flow is described in Fig. 1. A total of 14 patients were excluded due to impossibility for OCT imaging (pre-PCI = 2; post-PCI = 4) or low OCT imaging quality (pre-PCI = 3; post-PCI = 5). Baseline clinical characteristics are presented in Table 1. The population was predominantly male and characterized by a high prevalence of type 2 diabetes. Lesion characteristics (Table 2) were comparable among groups. Diameter stenosis as assessed by QCA was similar in the respective groups.

**Table 1:** Baseline characteristics of the study cohort (*n* = 84)

Clinical characteristics	Total study cohort *n* = 84
Age, years	70 (62–78)
Male	72.6% (61)
Diabetes	23.8 (20)
Hypertension	83.3% (70)
Non-smoker	26.2% (22)
Family history of CAD	50.0% (42)
Obesity	21.4%(18)
PAD	7.1% (6)
Prior ACS	32.1% (27)
Serum-Creatinine, mg/dl	1.0 (0.9–1.1)
Total Cholesterol, mg/dl	157 (131–191)
LDL, mg/dl	91 (69–128)

Data are expressed as median (IQR) or *n* (%).OCT denotes optical coherence tomography

*ACR* angiographic co-registration; *PAD* peripheral artery disease; *CAD* coronary artery disease; *ACS* acute coronary syndrome, *PCI* percutaneous coronary intervention, and *LDL* low-density lipoprotein

**Table 2:** Lesion characteristics

	Angio-guided *n* = 28	OCT-guided *n* = 28	ACR-guided *n* = 28	*p* for trend
Diameter stenosis, %	55.65 (51.05–67.60)	63.10 (59.10–66.50)	55.50 (48.30–67.35)	0.50
Minimal lumen diameter, mm	0.95 (0.80–1.10)	1.00 (0.80–1.30)	1.10 (0.90–1.30)	0.98
Lesion length, mm	17.25 (12.20–25.80)	17.90 (15.75–26.75)	18.40 (12.35–25.15)	0.54
Reference vessel diameter, mm	2.86 (2.51–3.23)	2.95 (2.61–3.27)	2.97 (2.35–3.36)	0.92
ACC/AHA-Type B2/C lesion	71.4% (20)	64.3% (18)	64.3% (18)	0.81
Bifurcation lesions*	25.0% (7)	28.6% (8)	25.0% (7)	0.94

*Lesion within true bifurcations (diameter side branch > 2.5). Data are expressed as median (IQR) or n (%). OCT denotes optical coherence tomography

*ACR* angiographic co-registration

### Primary endpoint

**Fig. 2:**
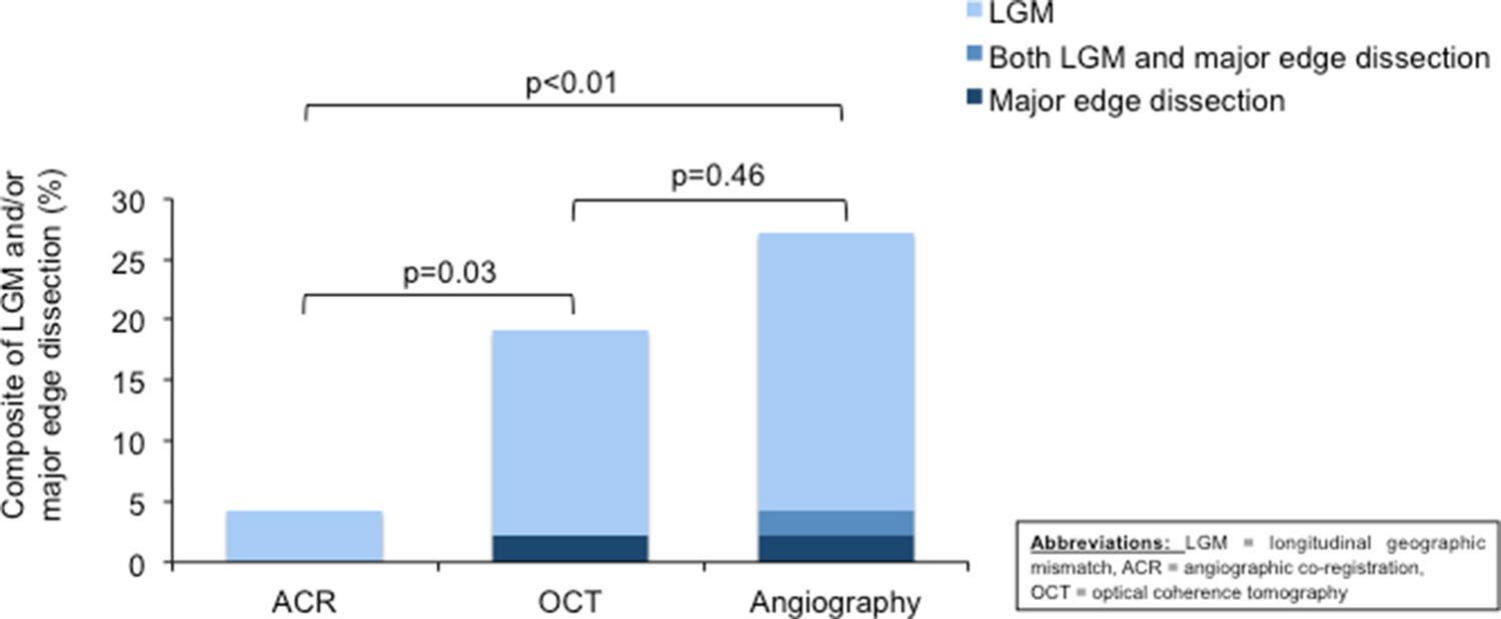
Primary endpoint of the composite consisting of major edge dissections and/or LGM. The primary endpoint was significantly lower in ACR group (4.2%, *n* = 2) compared to the OCT group (19.1%, *n* = 9, *p* = 0.03) and angiography group (25.5%, *n* = 12, *p* < 0.01). No significant difference was observed between the OCT and angiography group (*p* = 0.46). The light blue columns represent the incidence of longitudinal geographic mismatch. The middle blue columns represent the incidence of both longitudinal geographic mismatch and major edge dissection. The dark blue columns represent the incidence of major edge dissection. *ACR* denotes angiographic co-registration; *LGM* longitudinal geographic mismatch; *OCT* optical coherence tomography

The primary endpoint was significantly reduced by ACR-guided PCI (4.2%, *n* = 2) as compared to OCT-guided PCI (19.1%, *n* = 9, *p* = 0.03) and angiography-guided PCI (25.5%, *n* = 12, *p* < 0.01) (Fig. 2, Table 3). There was no significant difference between OCT- and angiography- guided PCI (*p* = 0.46).

**Table 3:** Incidence of primary and secondary study endpoints among the study groups

	Angio-guided *n* = 47 stent edges	OCT-guided *n* = 47 stent edges	ACR-guided *n* = 48 stent edges	Angio vs. OCT		ACR vs. OCT		ACR vs. Angio	
				OR (95% CI)	*p* value	OR (95% CI)	*p* value	OR (95% CI)	*p* value
Primary endpoint									
LGM and/or major edge dissection	25.5% (12/47)	19.1% (9/47)	4.2% (2/48)	1.45 (0.54–3.85)	0.46	0.18 (0.04–0.90)	0.03	0.13 (0.03–0.60)	< 0.01
Secondary endpoints									
LGM	22.9% (11/48)	17.0% (8/47)	4.2% (2/49)	1.45 (0.52–4.00)	0.47	0.21 (0.04–0.98)	0.04	0.14 (0.03–0.69)	0.02
Major edge dissection	4.3% (2/47)	2.1% (1/47)	0.0% (0/48)	2.04 (0.18–23.34)	0.57	–*	–*	–*	–*
Any edge dissection	25.5% (12/47)	17.4% (8/47)	0.0% (0/48)	1.63 (0.60–4.45)	0.34	–*	–*	–*	–*
Malapposition^1^	60.0% (30/50)	62.5% (30/48)	58.3% (28/48)	0.90 (0.28–2.87)	0.86	0.84 (0.26–2.70)	0.77	0.93 (0.30–2.94)	0.91
MSA proximal, mm^2#^	5.3 (4.3–7.0)	6.2 (5.4–7.6)	6.6 (4.4–7.6)	0.15 (− 0.72–2.20)	0.32	0.14 (− 0.80–2.07)	0.87	− 0.13 (− 1.04–0.31)	0.58
MSA distal, mm^2#^	5.0 (4.0–6.6)	4.9 (4.2–6.6)	4.7 (3.7–6.8)	0.09 (− 0.91–1.72)	0.66	0.14 (− 0.71–2.02)	0.35	− 0.08 (− 0.84–0.44)	0.55
Optimal or acceptable stent expansion^2^	62.5% (30/48)	68.0% (34/50)	82.6% (38/46)	0.78 (0.24–2.57)	0.69	2.24 (0.56–8.85)	0.25	2.85 (0.73–11.19)	0.13

Data are expressed as median (IQR) or *n* (%)

Stent struts clearly separated from the vessel wall without any tissue behind the struts with a distance from the adjacent intima of ≥ 0.2 mm and not associated with any side branch

^2^Optimal—MSA/Reference segment lumen area = ≥ 95%; acceptable—MSA/Reference segment lumen area =  ≥ 90% and < 95%

*OCT* denotes optical coherence tomography; *ACR* angiographic co-registration; *LGM* longitudinal geographical mismatch; *MSA* minimal stent area

*Not estimated because of small event numbers

^#^Instead of odds ratio regression coefficient given

### Secondary endpoints

Other predefined secondary endpoints are reported in Table 3. The incidence of LGM was significantly reduced with ACR- (4.2%) as compared to OCT- (17.0%; *p* = 0.04) and angiography-guided PCI (22.9%; *p* = 0.02). Major edge dissections affected the proximal edge in 13.9% of the cases and the distal edge in 14.3% of the cases. Whereas OCT-guided PCI was associated with reduced rates of any edge dissections after PCI as compared to angiography-guided PCI (17.4% vs. 25.5%; *p* = 0.34), no edge dissection was detectable after ACR-guided PCI. Malapposition as defined in this study occurred in 60.0%, 62.5% and 58.3% without a statistically significant difference, respectively. The stent results with respect to MSA and stent expansion were comparable among the three study groups (Table 3). This was also true for procedural characteristics (Table 4) as numbers of implanted stents, stent length, radiation exposure, amount of contrast medium and procedural time did not differ among groups (Table 4). Finally, in-hospital clinical complications were observed in two patients, one in the OCT (repeat coronary angiography due to recurrent chest pain) and one in the angiography group (acute coronary syndrome).

**Table 4:** Procedural characteristics among the three study groups

	Angio-guided *n* = 28	OCT-Guided *n* = 28	ACR-Guided *n* = 28	Angio vs. OCT *p* value	OCT vs. ACR *p* value	Angio vs. ACR *p* value
Procedure duration, min	53 (38–73)	49 (43–62)	49 (39–59	0.87	0.22	0.27
Fluoroscopy duration, min	13.67 (10.92–17.63)	14.05 (8.33–19.70)	13.73 (8.67–17.98)	0.98	0.80	0.79
Radiation dose, mGy	774.0 (525.0–895.0)	625.0 (442.5–894.0)	650 (381.5–885.5)	0.38	0.74	0.23
Contrast volume, ml	213.5 (160.0–248.0)	200.0 (165.0–246.0)	196.5 (159.0–222.5)	0.61	0.23	0.09
Stents per lesion	2 (1–2)	1 (1–2)	1 (1–2)	0.14	0.78	0.05
Max. stent diameter, mm	3.0 (2.75–3.5)	3.0 (3.0–3.5)	3.0 (2.5–3.5)	0.28	0.54	0.90
Stent length, mm	28 (18–38)	28 (23–35)	26 (18–33)	0.52	0.19	0.61

Data are expressed as median (IQR) or n (%). OCT denotes optical coherence tomography

ACR angiographic co-registration

### Predictors of longitudinal geographic missmatch and/or major edge dissection

Neither age, type 2 diabetes, nor acute coronary syndrome as index event or high lesion complexity (AHA class B2/C) was identified as significant predictors for the incidence of LGM or major edge dissections after PCI (Table 5). Importantly, the use of ACR- as compared to OCT-guided PCI [OR 0.17 (0.03–0.93); *p* = 0.04] as well as compared to angiographic-guided PCI [OR 0.12 (0.02–0.66); *p* = 0.02] was found to be inversely related with the primary endpoint (Table 5).

**Table 5:** Independent predictors on the primary combined study endpoint of LGM and/or major edge dissection

	Incidence of LGM and/or major edge dissection		
	OR	95% CI	*p* value
Angiographic vs. OCT-guided PCI	1.36	(0.49–3.75)	0.55
ACR-guided PCI vs. OCT-guided PCI	0.17	(0.03–0.93)	0.04
ACR-guided PCI versus Angiographic-guided PCI	0.12	(0.02–0.66)	0.02
Age	1.00	(0.96–1.05)	0.83
Diabetes mellitus	0.97	(0.33–2.84)	0.96
PCI within ACS-setting	0.40	(0.03–5.13)	0.48
ACC/AHA-Type B2/C lesion	1.83	(0.55–6.09)	0.32

ACR denotes angiographic co-registration

OCT optical coherence tomography; PCI Percutaneous coronary intervention; ACS acute coronary syndrome and ACC/AHA American College of Cardiology/American Heart Association

## Discussion

The OPTICO-integration II trial demonstrated for the first time a significant lower incidence of LGM and/or major edge dissections for ACR-guided PCIs as compared to an OCT- or angiography-guided approach.

Intracoronary imaging techniques revealed favorable results in terms of MACE as compared to angiography-guided PCI [10–12]. However, most of these studies investigated intravascular ultrasound (IVUS) and focused on stent optimization for complex coronary lesions [10–12]. Due to the limited axial resolution, especially in diffusely diseased calcified lesions, IVUS allows for advanced vessel sizing, without, however, providing detailed information on plaque composition and vessel-wall characteristics. Optical coherence tomography as a novel intracoronary high-resolution imaging technology with a tenfold increased axial resolution as compared to IVUS allows for more comprehensive lesion assessment. Its effect on PCI results as well as potential clinical benefits remains unknown. Recently, few clinical studies evaluating the effect of OCT guidance during PCI and including systematic OCT analysis during the phase of PCI planning were published [3, 5, 6, 13]. However, none of these studies specifically analyzed the implication of OCT-guided PCI planning on stent results [3, 5, 6, 13]. Not surprisingly, the incidence of untreated lesions in the reference segment was similar between OCT- and angiography-guided PCI in ILUMIEN III [5].

Since matching of the cross-sectional OCT images to the angiography by ACR became available [7], the optimal stent landing zone identified by OCT can be accurately translated to angiography during stent placement. A recent study demonstrated that ACR significantly changed the initial PCI strategy in almost half of the procedures, thereby improving the rate of complete lesion coverage, especially in complex coronary artery lesions prone to adverse events after PCI, e.g., diffusely diseased vessel segments or when precise stent deployment may be hampered due to missing anatomical landmarks [8].

In addition to a modification of the in PCI strategy by ACR, the current OPTICO-integration II trial for the first time demonstrates significantly improved PCI results by ACR with lower rates of LGM and edge dissections after PCI. A recent OCT study revealed incomplete lesion coverage after angiography-guided PCI in about 70% of the investigated lesions [14]. When real-time ACR was used, the frequency of LGM decreased by fivefold to less than 5% as this novel fusion imaging tool allowed for an exact localization of the intended stent landing zone compared to 17% longitudinal geographical mismatch in the OCT group without co-registration [14]. ACR guidance allows for an easy applicable and replicable correlation between the OCT-detected borders of the coronary lesion and its cross-sectional angiographic correlation [7], which emphasizes precise lesion coverage as revealed by the dramatically reduced LGM rate in this study. However, as stent length did not differ between ACR-, OCT- and angiography-guided PCI in this study, which is in line with other studies [5], the lower risk of LGM related to ACR may be explained mainly by a more accurate targeting of the stent landing zone rather than an extended lesion coverage.

Previous OCT studies identified high vulnerable plaque burden and severe circumferential calcifications at the stent edge as predictors of edge dissections after PCI [15, 16]. In the present study, stent edge dissection could be completely avoided when ACR was used, as compared to 17.4% when OCT-guidance was performed during PCI planning. Therefore, ACR may optimize stent sizing – including coverage of vulnerable plaques within the edges of the coronary lesions and optimal stent-positioning outside a vulnerable plaque to avoid incident edge dissections [16]. In a recent trial comparing ACR- with OCT-guided PCI, although no differences were observed in terms of LGM (27.6% with ACR vs. 34.0% with OCT; *p* = 0.33), a 50%-reduction in the risk of distal stent edge dissection after ACR-guided PCI was observed similar to this study [17]. Differences between the studies may at least in part be due different patient populations included and differences in the study protocols. In the present study, the primary endpoint was recorded immediately after achieving an angiographic optimal PCI result as compared to endpoint assessment after additional PCI-optimization by OCT or ACR, respectively, which may result in more frequent optimization procedures including additional stenting in case of inacceptable lesion coverage for OCT-guided PCI and consequently equalize potential differences between groups achieved by ACR [17].

Significant edge dissections after PCI [18] have been reported to be associated with a twofold increased risk of MACE [18–20] and likewise LGM is known to be a driver for target vessel revascularization and adverse events [21]. The large “Clinical Impact of OCT Findings During PCI” (CLI-OPCI II) trial pointed out a fivefold increased risk of MACE by uncovered residual plaques after PCI [3]. Although the present study was not designed and powered to detect differences in clinical outcomes, complete lesion coverage with simultaneously significant reduced rates of major edge dissections as achieved by ACR may positively affect procedural outcomes after PCI.

Several limitations need to be noted. Despite adequate patient size calculation and powering for the imaging endpoint in the present study, the total number of included patients is relatively low and allows no evaluation of potential impact on clinical endpoints. Furthermore, the present study is a single-center trial with all known limitations of this trial design. Since investigator blinding was not possible due to the study design, PCI results may have been influenced by operators’ individual preference and experience. However, an independent and blinded corelab analysis was performed to minimize this bias. The participating operators gained large experience regarding OCT-guided procedures in daily practice, which might influence OCT handling (e.g., correct and effective performance of OCT pullbacks) and stent implantation technique with respect to avoidance of PCI-related complications. This may explain the missing difference in contrast volume among the groups und low rate of edge dissections in all three study groups compared to other studies [5]. Moreover, endpoint analysis was based on the ILUMIEN IV-OPTIMAL PCI OCT-criteria. Investigators were trained for using these criteria in their OCT-guided PCI, which are considered the state-of-the-art of OCT guidance. However, the ongoing ILUMIEN IV- OPTIMAL PCI trial has yet to prove whether this approach has an impact on clinical outcomes.

## Conclusions

The OPTICO-integration II trial evaluated the effect of real-time optical coherence tomography co-registration with angiography on PCI results and demonstrated a significant reduction in the composite primary endpoint of LGM and/or major edge dissection—both relate with adverse outcomes following PCI—as compared to OCT- as well as to angiography-guided PCI, which was meanly driven by a reduction of LGM. The clinical value of this finding needs to be investigated in clinical outcome trials.

## Abbreviations

ACR: Angiographic co-registration
CCR: Chronic coronary syndrome
DES: Drug-eluting stents
eGFR: Estimated creatinine clearance
IQR: Interquartile range
IVUS: Intravascular ultrasound
LGM: Longitudinal geographic mismatch
LVEF: Left ventricular ejection fraction
MLA: Minimal lumen area
MSA: Minimal stent area
OCT: Optical coherence tomography
PCI: Percutaneous coronary interventions
QCA: Quantitative coronary angiography
STE-ACS: ST-segment elevation acute coronary syndromes

## References

[1] Neumann FJ, Sousa-Uva M, Ahlsson A, Alfonso F, Banning AP, Benedetto U (2019). 2018 ESC/EACTS Guidelines on myocardial revascularization. Eur Heart J.

[2] Windecker S, Stortecky S, Stefanini GG, da Costa BR, Rutjes AW, Di Nisio M (2014). Revascularisation versus medical treatment in patients with stable coronary artery disease: network meta-analysis. BMJ.

[3] Prati F, Romagnoli E, Burzotta F, Limbruno U, Gatto L, La Manna A (2015). Clinical impact of OCT findings during PCI: the CLI-OPCI II study. JACC Cardiovasc Imaging.

[4] Ino Y, Kubo T, Matsuo Y, Yamaguchi T, Shiono Y, Shimamura K (2016). Optical coherence tomography predictors for edge restenosis after everolimus-eluting stent implantation. Circ Cardiovasc Interv.

[5] Ali ZA, Maehara A, Genereux P, Shlofmitz RA, Fabbiocchi F, Nazif TM (2016). Optical coherence tomography compared with intravascular ultrasound and with angiography to guide coronary stent implantation (ILUMIEN III: OPTIMIZE PCI): a randomised controlled trial. Lancet.

[6] Meneveau N, Souteyrand G, Motreff P, Caussin C, Amabile N, Ohlmann P (2016). Optical coherence tomography to optimize results of percutaneous coronary intervention in patients with Non-ST-Elevation acute coronary syndrome: results of the multicenter, randomized DOCTORS study (does optical coherence tomography optimize results of stenting). Circulation.

[7] van der Sijde JN, Guagliumi G, Sirbu V, Shimamura K, Borghesi M, Karanasos A (2016). The OPTIS Integrated System: real-time, co-registration of angiography and optical coherence tomography. EuroIntervention.

[8] Leistner DM, Riedel M, Steinbeck L, Stahli BE, Frohlich GM, Lauten A (2017). Real-time optical coherence tomography coregistration with angiography in percutaneous coronary intervention-impact on physician decision-making: The OPTICO-integration study. Catheter Cardiovasc Interv.

[9] Roffi M, Patrono C, Collet JP, Mueller C, Valgimigli M, Andreotti F (2016). 2015 ESC Guidelines for the management of acute coronary syndromes in patients presenting without persistent ST-segment elevation: task force for the management of acute coronary syndromes in patients presenting without persistent ST-segment elevation of the european society of cardiology (ESC). Eur Heart J.

[10] Buccheri S, Franchina G, Romano S, Puglisi S, Venuti G, D'Arrigo P (2017). Clinical outcomes following intravascular imaging-guided versus coronary angiography-guided percutaneous coronary intervention with stent implantation: a systematic review and Bayesian network meta-analysis of 31 studies and 17,882 patients. JACC Cardiovasc Interv.

[11] Nerlekar N, Cheshire CJ, Verma KP, Ihdayhid AR, McCormick LM, Cameron JD (2017). Intravascular ultrasound guidance improves clinical outcomes during implantation of both first- and second-generation drug-eluting stents: a meta-analysis. EuroIntervention.

[12] Parise H, Maehara A, Stone GW, Leon MB, Mintz GS (2011). Meta-analysis of randomized studies comparing intravascular ultrasound versus angiographic guidance of percutaneous coronary intervention in pre-drug-eluting stent era. Am J Cardiol.

[13] Johnson TW, Raber L, di Mario C, Bourantas C, Jia H, Mattesini A (2019). Clinical use of intracoronary imaging. Part 2: acute coronary syndromes, ambiguous coronary angiography findings, and guiding interventional decision-making: an expert consensus document of the European Association of Percutaneous Cardiovascular Interventions. Eur Heart J.

[14] Hebsgaard L, Nielsen TM, Tu S, Krusell LR, Maeng M, Veien KT (2015). Co-registration of optical coherence tomography and X-ray angiography in percutaneous coronary intervention. the does optical coherence tomography optimize revascularization (DOCTOR) fusion study. Int J Cardiol.

[15] Wijns W, Shite J, Jones MR, Lee SW, Price MJ, Fabbiocchi F (2015). Optical coherence tomography imaging during percutaneous coronary intervention impacts physician decision-making: ILUMIEN I study. Eur Heart J.

[16] Chamie D, Bezerra HG, Attizzani GF, Yamamoto H, Kanaya T, Stefano GT (2013). Incidence, predictors, morphological characteristics, and clinical outcomes of stent edge dissections detected by optical coherence tomography. JACC Cardiovasc Interv.

[17] Koyama K, Fujino A, Maehara A, Yamamoto MH, Alexandru D, Jennings J (2019). A prospective, single-center, randomized study to assess whether automated coregistration of optical coherence tomography with angiography can reduce geographic miss. Catheter Cardiovasc Interv.

[18] Kobayashi N, Mintz GS, Witzenbichler B, Metzger DC, Rinaldi MJ, Duffy PL (2016). Prevalence, features, and prognostic importance of edge dissection after drug-eluting stent implantation: An ADAPT-DES intravascular ultrasound substudy. Circ Cardiovasc Interv.

[19] Prati F, Di Vito L, Biondi-Zoccai G, Occhipinti M, La Manna A, Tamburino C (2012). Angiography alone versus angiography plus optical coherence tomography to guide decision-making during percutaneous coronary intervention: the centro per la Lotta contro l'Infarto-optimisation of percutaneous coronary intervention (CLI-OPCI) study. EuroIntervention.

[20] Kang SJ, Cho YR, Park GM, Ahn JM, Kim WJ, Lee JY (2013). Intravascular ultrasound predictors for edge restenosis after newer generation drug-eluting stent implantation. Am J Cardiol.

[21] Costa MA, Angiolillo DJ, Tannenbaum M, Driesman M, Chu A, Patterson J (2008). Impact of stent deployment procedural factors on long-term effectiveness and safety of sirolimus-eluting stents (final results of the multicenter prospective STLLR trial). Am J Cardiol.

